# Type 2 Diabetes Mellitus and Kidney Cancer Risk: A Retrospective Cohort Analysis of the National Health Insurance

**DOI:** 10.1371/journal.pone.0142480

**Published:** 2015-11-11

**Authors:** Chin-Hsiao Tseng

**Affiliations:** 1 Department of Internal Medicine, National Taiwan University College of Medicine, Taipei, Taiwan; 2 Division of Endocrinology and Metabolism, Department of Internal Medicine, National Taiwan University Hospital, Taipei, Taiwan; 3 Division of Environmental Health and Occupational Medicine of the National Health Research Institutes, Zhunan, Taiwan; Massachusetts Eye & Ear Infirmary, Harvard Medical School, UNITED STATES

## Abstract

**Purpose:**

To evaluate the association between incidence of any kidney cancer and type 2 diabetes mellitus.

**Methods:**

A random sample of 1,000,000 subjects covered by the National Health Insurance was recruited. A total of 998728 people (115655 diabetes and 883073 non-diabetes) without kidney cancer at recruitment were followed from 2003 to 2005. The cumulative incidence of kidney cancer from 2003 to 2005 in diabetic patients and non-diabetic people in all ages and in age <40, 40–64, 65–74 and ≥75 years were calculated in the diabetic patients and the non-diabetic people, respectively. Logistic regression was used to estimate the odds ratios comparing diabetic patients to non-diabetic people in the respective age groups. Multivariable-adjusted odds ratios for kidney cancer with regards to diabetes status and diabetes duration (as a continuous variable or categorized into subgroups of non-diabetes, diabetes duration <1 year, 1–2.9 years, 3–4.9 years and ≥5 years) were estimated after multivariable adjustment. The multivariable-adjusted odds ratios for all baseline variables were also estimated for diabetic patients and non-diabetic people, respectively.

**Results:**

The 3-year cumulative incidence of kidney cancer in the diabetic patients and the non-diabetic people was 166.9 and 33.1 per 100,000 person-years, respectively. The incidence increased with regards to increasing age in both the diabetic patients and the non-diabetic people, but a higher risk of kidney cancer for the diabetic patients compared to the non-diabetic people was consistently observed in different age groups. After multivariable adjustment, the odds ratio for diabetic patients versus non-diabetic people was 1.7 (95% confidence interval: 1.3–2.1, *P*<0.01). While compared to the non-diabetic people, the odds ratio (95% confidence interval) for diabetes duration <1, 1–2.9 years, 3–4.9 years and ≥5 years was 1.5 (0.8–2.7), 1.6 (1.0–2.4), 1.6 (1.1–2.4) and 1.7 (1.3–2.3), respectively (*P*-trend <0.01). Analyses conducted in the diabetic patients and the non-diabetic people, respectively, consistently showed age, nephropathy and end-stage renal disease as significant risk factors of kidney cancer. Additionally, living in metropolitan Taipei region might also be associated with a higher risk of kidney cancer in the non-diabetic people, indicating a potential link between kidney cancer and some factors related to urbanization.

**Conclusions:**

Patients with type 2 diabetes mellitus have a significantly higher risk of kidney cancer.

## Introduction

Patients with type 2 diabetes mellitus suffer from a significantly higher risk of cancer involving the breast, endometrium, stomach, colorectum, liver, pancreas, urinary bladder, and lymphoid tissue [1–11]. The mechanisms of such an increased cancer risk in the diabetic patients may be related to insulin resistance, hyperinsulinemia, proinflammatory status and increased oxidative stress [1,12]. Although most studies conducted in western countries suggest a lower risk of prostate cancer in patients with type 2 diabetes mellitus [13], studies conducted in Taiwan and China do not conclude similarly and on the contrary support a higher risk of prostate cancer in patients with type 2 diabetes mellitus in terms of incidence [14,15], prevalence [16] and mortality [17]. Therefore, ethnical differences may exist when the risk of specific cancer types are compared between patients with diabetes mellitus and individuals without diabetes mellitus.

Kidney cancer is among the 10 most common cancers in the western world [18]. Its etiology remains unknown, but phenacetin-containing analgesics, smoking, obesity, hypertension, advanced kidney disease and genetic factors have been identified as potential risk factors [18–20]. On the other hand, alcohol consumption may be protective [18,19], especially in men [21]. Epidemiological studies investigating the association between kidney cancer risk and diabetes mellitus are not conclusive. As early as 1997, Wideroff et al. first reported a link between diabetes mellitus and kidney cancer in a population-based cohort of patients hospitalized with diabetes mellitus in Denmark [22]. The standardized incidence ratio (SIR) for kidney cancer comparing patients with diabetes mellitus to the general population was 1.4 (95% confidence interval: 1.2–1.6) for men, and 1.7 (95% confidence interval: 1.4–1.9) for women [22]. This was supported by a later study published in 1999 by Lindblad et al., who compared the incidences of kidney cancer in patients with diabetes mellitus identified in the Swedish Inpatient Register to the general population [23]. The SIR in men was 1.3 (95% confidence interval: 1.1–1.6), and 1.7 (95% confidence interval: 1.4–2.0) in women [23]. On the other hand, the link between kidney cancer risk and diabetes mellitus can not be similarly shown by some other later observational studies. For example, Setiawan et al. followed a multiethnic cohort in Hawaii and Los Angeles for up to 8.3 years, and they estimated a relative risk of 1.1 (95% confidence interval: 0.8–1.6) for men, and 1.2 (95% confidence interval: 0.7–1.9) for women [21]. In another multicenter case-control study conducted in Italy, Zucchetto et al. estimated an odds ratio of 1.3 (95% confidence interval: 0.9–1.7) for a history of diabetes mellitus [12]. Two independent meta-analyses published recently suggest a pooled relative risk (95% confidence interval) of 1.4 (1.1–1.9) derived from 9 cohort studies [24] and 1.4 (1.2–1.7) from 24 studies [25].

Most of the studies included in the two meta-analyses were conducted in western populations and only four studies in the meta-analyses were conducted in Asian populations, three with a cohort design [26–28] and one with a case-control design [29]. Except for one study being conducted in the Korean population [28], the other three were conducted in the Japanese [26,27,29]. Although a summary relative risk derived from the four Asian studies was significant: 1.3 (1.1–1.6) [25], none of the individual studies showed a significantly higher risk of kidney cancer in the diabetic patients, except for one Japanese study showing a significantly higher risk only for men but not for women [26]. There are some limitations associated with these previous Asian studies: 1) They were not population-based; 2) They did not differentiate type 1 and type 2 diabetes mellitus; 3) Diabetes mellitus was self-reported; and 4) The case numbers of kidney cancer ranged from 37 [27] to 134 [26] were too small for evaluating the effect of subgroups of diabetes duration.

Therefore, the association between diabetes mellitus and kidney cancer risk requires further investigation, especially in the Asian populations. The purpose of the present study was to evaluate the association between the incidence of any kidney cancer and type 2 diabetes mellitus in the Chinese population in Taiwan by using the National Health Insurance (NHI) reimbursement databases.

## Materials and Methods

This study was approved by an ethics review board of the National Health Research Institutes with an approval number 99274. The National Health Research Institutes is the only organization approved, as per local regulations, for handling the NHI reimbursement database for academic research. For the protection of privacy, the identification information of individuals was scrambled and the patient records/information was anonymized and de-identified prior to analysis. Written informed consent from the participants to use their clinical records in the study was not obtained, because this is not required according to local regulations. The analyses and reporting of the study were conducted according to the STROBE guidelines [30,31], by using the checklist for observational studies (version 4) [32].

This is a retrospective cohort study using the reimbursement databases of the NHI and the methods used in this study have been described in detail previously where a different endpoint of prostate cancer was investigated [14]. In brief, the longitudinal reimbursement databases of a random sample of 1,000,000 subjects covered by the NHI in 2005 were retrieved. The databases were available back to 1996 and diabetes mellitus was coded 250.XX and kidney cancer 189, based on the *International Classification of Diseases*, *Ninth Revision*, *Clinical Modification* (ICD-9-CM).

After excluding type 1 diabetes mellitus (in Taiwan, patients with type 1 diabetes mellitus were issued a so-called “Severe Morbidity Card” after certified diagnosis and they are waived for much of the co-payments), living region unknown, and kidney cancer diagnosed before 2003, 115,655 patients with type 2 diabetes mellitus and 883,073 individuals without diabetes mellitus were followed from the beginning of 2003 to the end of 2005.

Baseline characteristics between patients with type 2 diabetes mellitus and non-diabetic people were compared by Student’s t test for age, and by Chi square test for sex and the following categories of variables: comorbidities, medications, living region and occupation. The comorbidities included hypertension, chronic obstructive pulmonary disease (a surrogate for smoking), stroke, nephropathy, ischemic heart disease, peripheral arterial disease, eye disease related to diabetes mellitus [including the following diagnoses (ICD-9-CM code): diabetes with ophthalmic manifestations (250.5), diabetic retinopathy (362.0), blindness and low vision (369), diabetic cataract (366.41) and glaucoma associated with systemic syndromes (365.44)], obesity and dyslipidemia as detailed elsewhere [14]. In consideration that smoking and end-stage renal disease [18–20] are important risk factors of kidney cancer, but alcohol consumption may be protective [18,19], diagnosis of tobacco abuse (305.1, 649.0, 989.84), end-stage renal disease (585, 403.01, 403.11, 403.91, 404.02, 404.03, 404.12, 404.13, 404.92, 404.93) and alcohol-related diagnoses (291, 303, 535.3, 571.0–571.3, 980.0) were additionally retrieved from the database and included as covariates. Medications included sulfonylurea, metformin, insulin, acarbose, pioglitazone, rosiglitazone, angiotensin-converting enzyme inhibitor and/or angiotensin receptor blocker, calcium channel blocker, statin and fibrate. Living region was categorized as Taipei, Northern, Central, Southern, and Kao-Ping and Eastern; and occupation was categorized as I: civil servants, teachers, employees of governmental or private business, professionals and technicians; II: people without particular employers, self-employed or seamen, III: farmers or fishermen; and IV: low-income families supported by social welfare or veterans [14].

The cumulative incidences of kidney cancer from 2003–2005 in diabetic patients and non-diabetic people were calculated for all ages and for age <40, 40–64, 65–74 and ≥75 years, respectively. Unadjusted odds ratios and their 95% confidence intervals for kidney cancer comparing diabetic patients versus non-diabetic people were then estimated by logistic regression. Sensitivity analyses were also performed by excluding patients with diabetes duration <5 years to minimize the possibility that diabetes mellitus might be caused by kidney cancer.

To evaluate whether diabetes status (yes versus no) or diabetes duration (as a continuous variable, or categorized as <1, 1–2.9, 3–4.9 and ≥5 years versus non-diabetes) might be associated with kidney cancer after multivariable adjustment, odds ratios and their 95% confidence intervals for kidney cancer were estimated by logistic regression with all baseline characteristics entered as independent variables.

Additional logistic regression models were created to estimate the multivariable-adjusted odds ratios and their 95% confidence intervals for all independent variables in the diabetic patients and in the non-diabetic people, separately. In the database, it was noted that, during the study period, sulfonylurea and metformin were the two classes of antidiabetic drugs most commonly used in Taiwan, and the use of them was highly correlated with a calculated correlation coefficient of 0.665 (*P*<0.01). To avoid the problem of hypercollinearity, the use of sulfonylurea and/or metformin was treated as a single independent variable.

Analyses were conducted using SAS statistical software, version 9.3 (SAS Institute, Cary, NC). *P*<0.05 was considered as statistically significant.

## Results

The baseline characteristics between the diabetic patients and the non-diabetic people are compared in Table 1. All of the variables differed significantly between the two groups. The diabetic patients were characterized by older age, female predominance, having more comorbidities, higher prevalent rates of taking medications, more living in Southern and Kao-Ping and Eastern regions, and less involvement in occupation class I (i.e., civil servants, teachers, employees of governmental or private business, professionals and technicians). The mean diabetes duration in the diabetic patients was 5.8 years.

**Table 1:** Baseline characteristics of study subjects with and without diabetes mellitus.

Variables	Diabetes mellitus				*P*
	No		Yes		
	*n*	%	*n*	%	
*n* = 998703	883053		115650		
Age (years)*	33.±19.4		57.3±16.7		<0.01
Sex (men)	442716	50.1	52398	45.3	<0.01
Diabetes duration (years)*	-	-	5.8±3.0		
Hypertension	97351	11.0	66228	57.3	<0.01
Chronic obstructive pulmonary disease	202392	22.9	50371	43.6	<0.01
Stroke	34309	3.9	26555	23.0	<0.01
Nephropathy	36230	4.1	24008	20.8	<0.01
End-stage renal disease	1310	0.2	1798	1.6	<0.01
Ischemic heart disease	54244	6.1	40501	35.0	<0.01
Peripheral arterial disease	23621	2.7	18339	15.9	<0.01
Eye disease	2513	0.3	11791	10.2	<0.01
Obesity	5574	0.6	2932	2.5	<0.01
Dyslipidemia	65573	7.4	59231	51.2	<0.01
Tobacco abuse	4512	0.5	1109	1.0	<0.01
Alcohol-related diagnoses	10950	1.2	4313	3.7	<0.01
Statin	21105	2.4	28803	24.9	<0.01
Fibrate	18257	2.1	24126	20.9	<0.01
Angiotensin-converting enzyme inhibitor/angiotensin receptor blocker	61855	7.0	51319	44.4	<0.01
Calcium channel blocker	72953	8.3	51431	44.5	<0.01
Sulfonylurea	-	-	47451	41.0	
Metformin	-	-	41344	35.8	
Insulin	-	-	10350	9.0	
Acarbose	-	-	8909	7.7	
Pioglitazone	-	-	2874	2.5	
Rosiglitazone	-	-	8313	7.2	
Living region					
Taipei	321410	36.4	39518	34.2	<0.01
Northern	128587	14.6	14304	12.4	
Central	163711	18.5	19634	17.0	
Southern	119860	13.6	19872	17.2	
Kao-Ping and Eastern	149485	16.9	22322	19.3	
Occupation category					
I	484736	54.9	43904	38.0	<0.01
II	144075	16.3	21675	18.7	
III	113975	12.9	26271	22.7	
IV	140267	15.9	23800	20.6	

*Age and diabetes duration are expressed as mean ± standard deviation

Refer to Materials and Methods for the categories of occupation

The 3-year cumulative incidences of kidney cancer and the unadjusted odds ratios for kidney cancer for the diabetic patients versus the non-diabetic people in all ages and in different age groups are shown in Table 2. It is evident that the diabetic patients consistently showed a significantly higher risk of kidney cancer than people without diabetes mellitus disregarding age. The findings were consistently demonstrated in the sensitivity analyses when patients with diabetes mellitus diagnosed for <5 years were excluded (except for a higher risk without statistical significance in the age group of <40 years).

**Table 2:** Cumulative incidence (per 100,000) of kidney cancer from 2003 to 2005 in diabetic patients and non-diabetic people by age and the unadjusted odds ratios comparing diabetic patients to non-diabetic people.

Age	Type 2 diabetes mellitus				Odds ratio (95% confidence interval)	*P*
	Yes		No			
	*n* / *N*	3-year cumulative incidence	*n* / *N*	3-year cumulative incidence		
**Diabetes mellitus of any duration**						
All ages	193 / 115655	166.9	292 / 883073	33.1	5.1 (4.2–6.1)	<0.01
Age <40 years	8 / 17270	46.3	42 / 570472	7.4	6.3 (3.0–13.4)	<0.01
Age 40–64 years	57 / 57199	99.7	147 / 253826	57.9	1.7 (1.3–2.3)	<0.01
Age 65–74	60 / 23181	258.8	60 / 34486	174.0	1.5 (1.0–2.1)	<0.05
Age ≥75	68 / 18005	377.7	43 / 24289	177.0	2.1 (1.5–3.1)	<0.01
**Sensitivity analysis after excluding patients with diabetes mellitus diagnosis for <5 years**						
All ages	129 / 69494	185.6	292 / 883073	33.1	5.6 (4.6–6.9)	<0.01
Age <40 years	2 / 8586	23.3	42 / 570472	7.4	3.2 (0.8–13.1)	0.11
Age 40–64 years	34 / 32361	105.1	147 / 253826	57.9	1.8 (1.3–2.6)	<0.01
Age 65–74	41 / 15716	260.9	60 / 34486	174.0	1.5 (1.0–2.2)	<0.05
Age ≥75	52 / 12831	405.3	43 / 24289	177.0	2.3 (1.5–3.4)	<0.01

*n*: case number of kidney cancer, *N*: case number observed

Table 3 shows the multivariable-adjusted odds ratios for kidney cancer with regards to diabetes status and diabetes duration. Diabetes status is significantly associated with a higher risk of kidney cancer after multivariable adjustment, with an odds ratio of 1.7 (95% confidence interval 1.3–2.1). For every 1-year increment of diabetes duration, the risk increased approximately 10% (odds ratio 1.1, 95% confidence interval 1.0–1.1, *P*<0.01). When diabetes duration was categorized into subgroups, the odds ratio increased from 1.5 in patients with diabetes duration <1 year to 1.6 in patients with diabetes duration ranged from 1 to 4.9 years, and to 1.7 in patients with diabetes duration ≥5 years (*P*-trend <0.01).

**Table 3:** Multivariable-adjusted odds ratios for kidney cancer with regards to diabetes status and diabetes duration (either treated as a continuous variable or categorized into subgroups).

Variables	Interpretation	Odds ratio*	95% confidence interval	*P*
Diabetes status	No	1.0		
	Yes	1.7	(1.3–2.1)	<0.01
Diabetes duration	Every 1-year increment	1.1	(1.0–1.1)	<0.01
Diabetes duration	Non-diabetes	1.0		
	Diabetes duration <1 year	1.5	(0.8–2.7)	0.23
	Diabetes duration 1–2.9 years	1.6	(1.0–2.4)	0.04
	Diabetes duration 3–4.9 years	1.6	(1.1–2.4)	0.03
	Diabetes duration ≥5 years	1.7	(1.3–2.3)	<0.01
				*P*-trend <0.01

*Adjusted for all variables in Table 1

Table 4 shows the odds ratios for all independent variables in the logistic regression models conducted in the diabetic patients and the non-diabetic people, respectively. It is evident that age, nephropathy and end-stage renal disease were significant risk factors for both the diabetic patients and the non-diabetic people. None of the other variables was significantly associated with kidney cancer in the diabetic patients, but living region was additionally associated with kidney cancer in the non-diabetic people. People living in regions other than the most metropolitan Taipei city seemed to have a lower risk.

**Table 4:** Multivariable-adjusted odds ratios for kidney cancer with regards to diabetes status.

Variables	Interpretation	*n* / *N*	Diabetes mellitus			*n* / *N*	Non-diabetes		
			OR	95% CI	*P*		OR	95% CI	*P*
				*N* = 115650				*N* = 883053	
Age	Every 1-year increment	485 / 998703	1.1	(1.0–1.1)	<0.01	435 / 410976	1.1	(1.0–1.1)	<0.01
Sex	Men vs. Women	85 / 52398; 108 / 63252	0.9	(0.7–1.2)	0.50	153 / 442716; 139 / 440337	1.1	(0.9–1.4)	0.52
Diabetes duration	Every 1-year increment	485 / 998703	1.0	(1.0–1.1)	0.14	-	-	-	-
Hypertension	Yes vs. No	133 / 66228; 60 / 49422	1.0	(0.6–1.5)	0.92	117 / 97351; 175 / 785702	1.0	(0.7–1.5)	0.98
COPD	Yes vs. No	94 / 50371; 99 / 65279	0.9	(0.6–1.2)	0.36	92 / 202392; 200 / 680661	0.8	(0.6–1.1)	0.13
Stroke	Yes vs. No	56 / 26555; 137 / 89095	0.8	(0.6–1.1)	0.17	40 / 34309; 252 / 848744	0.8	(0.5–1.1)	0.22
Nephropathy	Yes vs. No	74 / 24008; 119 / 91642	1.6	(1.1–2.2)	0.01	86 / 36230; 206 / 846823	3.5	(2.6–4.7)	<0.01
End-stage renal disease	Yes vs. No	20 / 1798; 173 / 113852	6.1	(3.6–10.6)	<0.01	22 / 1310; 270 / 881743	8.3	(5.0–13.7)	<0.01
Ischemic heart disease	Yes vs. No	89 / 40501; 104 / 75149	1.1	(0.8–1.5)	0.69	69 / 54244; 223 / 828809	1.0	(0.7–1.4)	0.93
Peripheral arterial disease	Yes vs. No	37 / 18339; 156 / 97311	0.9	(0.6–1.2)	0.40	26 / 23621; 266 / 859432	1.0	(0.6–1.5)	0.84
Eye disease	Yes vs. No	24 / 11791; 169 / 103859	1.0	(0.6–1.6)	0.84	0 / 2513; 292 / 880540	-	-	-
Obesity	Yes vs. No	5 / 2932; 188 / 112718	1.8	(0.7–4.3)	0.22	3 / 5574; 289 / 877479	1.5	(0.5–4.6)	0.51
Dyslipidemia	Yes vs. No	100 / 59231; 93 / 56419	1.0	(0.7–1.4)	0.96	65 / 65573; 227 / 817480	1.2	(0.8–1.6)	0.39
Tobacco abuse	Yes vs. No	1 / 1109; 192 / 114541	0.7	(0.1–5.2)	0.74	0 / 4512; 292 / 878541	-	-	-
Alcohol-related diagnoses	Yes vs. No	7 / 4313; 186 / 111337	1.3	(0.6–2.8)	0.48	8 / 10950; 284 / 872103	1.5	(0.7–3.0)	0.29
Statin	Yes vs. No	50 / 28803; 143 / 86847	0.9	(0.6–1.4)	0.73	27 / 21105; 265 / 861948	0.9	(0.5–1.4)	0.55
Fibrate	Yes vs. No	40 / 24126; 153 / 91524	0.9	(0.6–1.3)	0.63	24 / 18257; 268 / 864796	1.2	(0.7–1.9)	0.52
ACEI/ARB	Yes vs. No	104 / 51319; 89 / 64331	0.9	(0.6–1.4)	0.71	85 / 61855; 207 / 821198	1.1	(0.8–1.6)	0.65
Calcium channel blocker	Yes vs. No	102 / 51431; 91 / 64219	0.7	(0.5–1.1)	0.11	91 / 72953; 201 / 810100	1.0	(0.7–1.4)	0.82
Sulfonylurea/metformin	Yes vs. No	89 / 53708; 104 / 61942	1.0	(0.7–1.4)	0.99	-	-	-	-
Insulin	Yes vs. No	19 / 10350; 174 / 105300	0.7	(0.4–1.3)	0.26	-	-	-	-
Acarbose	Yes vs. No	9 / 8909; 184 / 106741	0.6	(0.3–1.2)	0.14	-	-	-	-
Pioglitazone	Yes vs. No	3 / 2874; 190 / 112776	0.8	(0.2–2.5)	0.65	-	-	-	-
Rosiglitazone	Yes vs. No	10 / 8313; 183 / 107337	0.8	(0.4–1.6)	0.48	-	-	-	-
Living region	Northern vs. Taipei	18 / 14304; 63 / 39518	0.8	(0.5–1.3)	0.34	23 / 128587; 134 / 321410	0.5	(0.3–0.7)	<0.01
	Central vs. Taipei	31 / 19634; 63 / 39518	1.0	(0.7–1.6)	0.84	45 / 163711; 134 / 321410	0.7	(0.5–0.9)	0.02
	Southern vs. Taipei	42 / 19872; 63 / 39518	1.4	(0.9–2.1)	0.14	42 / 119860; 134 / 321410	0.8	(0.5–1.1)	0.17
	Kao-Ping/Eastern vs. Taipei	39 / 22322; 63 / 39518	1.2	(0.8–1.8)	0.45	48 / 149485; 134 / 321410	0.7	(0.5–1.0)	0.03
Occupation	II vs. I	29 / 21675; 64 / 43904	0.9	(0.6–1.5)	0.81	58 / 144075; 125 / 484736	1.2	(0.9–1.6)	0.30
	III vs. I	51 / 26271; 64 / 43904	0.8	(0.5–1.1)	0.18	57 / 113975; 125 / 484736	0.9	(0.6–1.3)	0.55
	IV vs. I	49 / 23800; 64 / 43904	0.9	(0.6–1.3)	0.60	52 / 140267; 125 / 484736	0.8	(0.6–1.1)	0.15

*n*: case number of kidney cancer, *N*: case number observed

OR: odds ratio, CI: confidence interval, COPD: chronic obstructive pulmonary disease, ACEI/ARB: angiotensin-converting enzyme inhibitor/angiotensin receptor blocker

Refer to Materials and Methods for the categories of occupation

## Discussion

This is the first study showing a definitely higher risk of kidney cancer in patients with known type 2 diabetes mellitus in the Chinese population in Taiwan (Tables 2 and 3). Furthermore, there seems to be a dose-response relationship between diabetes duration and kidney cancer risk when both diabetic patients and non-diabetic people were analyzed together (Table 3), though this could not be similarly demonstrated when only diabetic patients were analyzed (Table 4). Kidney cancer is associated with a poor prognosis and nearly half of the patients die within 5 years after diagnosis [33]. In the present study, diabetes mellitus was unlikely caused by kidney cancer, because the association was consistently observed when patients with a diagnosis of diabetes mellitus <5 years were excluded in the analyses (Table 2), taking into account that diabetes mellitus diagnosed more than 5 years before kidney cancer can hardly be a consequence of the carcinogenic process.

The present study confirmed the findings of a significantly higher risk of kidney cancer in patients with diabetes mellitus observed in two independent meta-analyses, both of which demonstrated a pooled relative risk of 1.4 [24,25]. In the meta-analyses, there are four studies evaluating the association between diabetes mellitus and kidney cancer risk in the Asian populations, specifically Koreans and Japanese [26–29]. However, none of them were conducted at the population level and they did not differentiate type 2 diabetes mellitus from type 1 diabetes mellitus. Because type 1 diabetes mellitus may not be associated with kidney cancer risk in one study [34] (this may require confirmation, especially in patients with type 1 diabetes mellitus and chronic kidney disease), a mixture of both types of diabetes mellitus might have attenuated the true association. Most of these previous Asian studies recruited self-reported diabetes mellitus, which could also have underestimated the relative risk for kidney cancer because many patients with diabetes mellitus might not have reported as having the disease. The small case numbers of kidney cancer (ranged from 37 to 134) in these studies could also explain a lack of significant association in the studies.

In a recently published population-based case-control study conducted in Taiwan, diabetes mellitus was not associated with kidney cancer risk in either the unadjusted or adjusted models [35]. Although this study also used the NHI database, it should be pointed out that the investigators used a different approach of case-control design by including small numbers of 116 cases with kidney cancer and 464 controls without kidney cancer. There were only 68 and 21 patients with diabetes mellitus in the cases and controls, respectively. Therefore, this study might be underpowered to detect a significant association between type 2 diabetes mellitus and kidney cancer. On the other hand, another recent study estimating the SIR for kidney cancer comparing patients with diabetes mellitus to the general population in Taiwan suggested a significantly higher risk of kidney cancer in the diabetic patients. The estimated SIR (95% confidence interval) was 1.32 (1.25–1.40) and 1.38 (1.30–1.46) in men and women, respectively [36]. However, this study did not consider the adjustment for any potential confounders.

There are also several studies estimating the SIR in other countries after the publication of the two meta-analyses [24,25]. The findings remained inconclusive. For example, a study conducted in China suggested a significantly higher risk of kidney cancer in the diabetic patients [SIR: 1.6 (1.3–2.0) in men and 1.7 (1.3–2.3) in women] [15]. This was similarly shown in studies conducted in Sweden [37] and Australia [38], but not in another study conducted in Tyrol, Austria [39]. The use of the general population, which may also include patients with diabetes mellitus, as the standard in the calculation of SIR and the lack of individual information on potential confounders are major limitations in these studies using SIR as an indicator.

The present study has merits to overcome all of these limitations. First, because the NHI is a universal health system and covers more than 98% of the total population, the present study basically included a population-based sample from the whole nation. Second, patients with type 1 diabetes mellitus had been excluded from the study and therefore, only patients with type 2 diabetes mellitus were included for analyses. Because type 1 diabetes mellitus is considered a severe morbidity in the NHI and most of the copayments can be waived for the patients after certified diagnosis, most patients with type 1 diabetes mellitus were holding such a Severe Morbidity Care with the diagnosis. Although the sensitivity and specificity of using “Severe Morbidity Card” as a surrogate for the diagnosis of type 1 diabetes mellitus have not been evaluated previously, both are believed to be good for the following reasons. Patients with type 1 diabetes mellitus would tend to request for issuing such a card because they can be waived for many of the copayments that patients with type 2 diabetes mellitus would not be privileged. However, such a card would not be issued until after a diagnosis is confirmed by the history of diabetes onset with ketoacidosis together with laboratory examinations of C-peptide and/or glucagon test. Furthermore, because the incidence of type 1 diabetes mellitus in Taiwan is very low (approximately 4 per 100,000 per year [40]), they represent only a small proportion (<1% [41]) of the total diabetes population. The impact of misclassification in such a small proportion of the diabetic patients is expected to be trivial. Third, the large number of kidney cancer cases (*n* = 485) rendered a possibility to analyze the dose-response relationship with subgroups of diabetes duration (Table 3). Fourth, the present study could discern patients with diabetes mellitus and people without diabetes mellitus in the population and therefore reduce the potential bias from a mixture of diabetes and non-diabetes in the standard population as seen in studies using SIR. Additionally, though not absolutely accurate, some potential confounders could be adjusted for by including the diagnosis of comorbidities and used medications in the modeling.

Advanced kidney disease has been identified as an important risk factor of kidney cancer [18,19]. This is consistently shown in previous studies conducted in the Chinese people living either in China [20] or in Taiwan [35]. In the present study, besides age, nephropathy and end-stage renal disease were the two most important risk factors of kidney cancer in either the diabetic patients or the non-diabetic people (Table 4). The consistent demonstration of a close link between kidney cancer and these important risk factors indicated the validity of the study.

According to the estimation of the International Diabetes Federation, diabetes mellitus is on the rise and there are 382 million people with diabetes mellitus around the world in 2014. China is the most populated country with diabetes mellitus (*n* = 98.4 million) [42], and thus an elucidation of a link between type 2 diabetes mellitus and kidney cancer risk is especially important in the Chinese population. For the Chinese living in Taiwan, the incidence of type 2 diabetes mellitus has been increasing [43], but this was not similarly observed for type 1 diabetes mellitus [40]. In Taiwan, in parallel with the increasing trend of diabetes mellitus, the age-standardized incidence rate of kidney cancer also increased from 3.2 per 100,000 population in 1980–1984 to 7.4 per 100,000 in 2000–2006 [44]. If the findings in the present study can be applied to the Chinese population living on mainland China, the global impact of diabetes mellitus on kidney cancer incidence can be huge.

The study by Zucchetto et al. suggested that the link between diabetes mellitus and renal cell carcinoma may be through hypertension [12]. However, they failed to adjust for the most important confounder of chronic kidney disease. Since hypertension is closely linked to chronic kidney disease, it could not be excluded that the link between diabetes mellitus and renal cell carcinoma might also be ascribed to the effect of chronic kidney disease.

Smoking has been identified as an important risk factor of kidney cancer in some studies [45], but this was not confirmed in a study conducted in China [20]. In the present study, because the information of smoking was not available, only chronic obstructive pulmonary disease and tobacco abuse could be used as surrogates. Both of them did not show a significant association with kidney cancer in either the diabetic patients or the non-diabetic people (Table 4). Because the validity of using these surrogate diagnoses has not been evaluated previously, residual confounding from smoking could not be completely excluded. It is true that patients with such diagnoses might represent only a small proportion of very heavy smokers who developed clinical diseases and therefore exposure misclassification was expected. If such exposure misclassification was not related to kidney cancer (nondifferential), the estimated effect would be expected to bias toward the null [46]. On the other hand, if the exposure misclassification was differential, the true association could either be overestimated or underestimated [46]. The lack of association between kidney cancer and smoking, alcohol consumption and occupation in the present study (Table 4) actually all echoed the findings of a previous study conducted in the Chinese people living in China [20].

People living in the most metropolitan Taipei city seemed to have a higher risk of kidney cancer than people living in other regions in Taiwan (Table 4), suggesting that lifestyle, socioeconomic status or some environmental exposure related to urbanization may play some role in the development of kidney cancer. This has been similarly demonstrated in early studies conducted in the US [47], Italy [48] and Taiwan [49], showing that kidney cancer mortality was associated with urbanization and socioeconomic status. However, this could only be demonstrated in people without diabetes mellitus but not in the diabetic patients (Table 4). One explanation for such a lack of association between living region and kidney cancer in the diabetic patients might be due to the entanglement between diabetes mellitus and factors related to urbanization and socioeconomic status, leading to an observation of a close link between diabetes mellitus and kidney cancer but a diminished association between living region and kidney cancer after multivariable adjustment in the diabetic patients (Tables 2 and 3).

Although the present study reinforced diabetes mellitus as a risk factor of kidney cancer in Taiwan, whether such an association can be generalized to the large numbers of diabetic patients living in other Asian countries (e.g., China or India) or in the middle east countries remains to be confirmed. Since diabetes mellitus [42] and kidney cancer [19] are both increasing worldwide, clarification of such an association may have great impact on the prevention of both diseases. With successful lifestyle modification to prevent type 2 diabetes mellitus, the increasing incidence of kidney cancer may also be attenuated.

There are several strengths in this study. First, the analyses were based on big data with a large sample size from the whole nation. The large sample size and the massive amount of data allowed the exclusion of type 1 diabetes mellitus for evaluating the association of kidney cancer in a more homogeneous group of patients with type 2 diabetes mellitus with the consideration of adjustment for some important risk factors and the evaluation of the dose-response effect with regards to diabetes duration. Second, the database included reimbursement information from outpatients and inpatients and the diagnoses were caught from both sources. Third, cancer is regarded as a severe morbidity by the Bureau of NHI and those with a certified diagnosis of cancer can be waived for most medical co-payments. Therefore the detection rate would not tend to be affected by different social classes. Fourth, the use of medical record might have reduced the potential bias related to self-reporting. Fifth, the massive logistic regression conducted in the present study not only consistently demonstrated a close link between type 2 diabetes mellitus and kidney cancer risk, the validity of the study could also be ascertained by the demonstration of a close link between kidney cancer and well recognized risk factors such as age, nephropathy and end-stage renal disease.

Limitations of the study included a lack of information on the pathology, grading and staging of kidney cancer. However, because more than 90% of all cases of kidney cancer in Taiwan belong to renal cell carcinoma [50], it is believed that most cases of kidney cancer referred to in the present study should be of renal cell carcinoma. Second, the study did not have information of actual measurement of confounders such as anthropometric parameters for obesity, family history, diet, lifestyle, physical activity, smoking, alcohol drinking, hormones and genetic parameters. Third, biochemical data such as lipid profile, glucose, insulin and C-peptide were not available for investigation. Finally, the follow-up interval might be too short for the likely induction time between diabetes mellitus and kidney cancer.

In summary, this population-based and large sample sized analysis of the NHI reimbursement databases suggests that patients with type 2 diabetes mellitus in Taiwan have an increased risk of kidney cancer when compared to people without diabetes mellitus. Because both the incidences of kidney cancer [19,44] and type 2 diabetes mellitus [43] are increasing, the impact of kidney cancer on the population, especially those with type 2 diabetes mellitus, should warrant public health attention. However, future studies are required to elucidate whether patients with type 1 diabetes mellitus may also have a higher risk of kidney cancer, especially in the presence of chronic kidney disease. More detailed investigation on the potential impacts of different classes of antidiabetic drugs and disease severity on the link between diabetes mellitus and kidney cancer are important issues worthy of future studies. Finally, because the distribution of potential confounders may vary among different ethnicities, whether the elevated risk of kidney cancer in association with diabetes mellitus observed in the present study can be generalized to other ethnicities remains to be answered.
